# Mitochondrial Carriers Link the Catabolism of Hydroxyaromatic Compounds to the Central Metabolism in *Candida parapsilosis*

**DOI:** 10.1534/g3.116.034389

**Published:** 2016-10-03

**Authors:** Igor Zeman, Martina Neboháčová, Gabriela Gérecová, Kornélia Katonová, Eva Jánošíková, Michaela Jakúbková, Ivana Centárová, Ivana Dunčková, L'ubomír Tomáška, Leszek P. Pryszcz, Toni Gabaldón, Jozef Nosek

**Affiliations:** * Department of Biochemistry, Comenius University in Bratislava, Faculty of Natural Sciences, 842 15, Slovak Republic; † Department of Genetics, Comenius University in Bratislava, Faculty of Natural Sciences, 842 15, Slovak Republic; ‡ Bioinformatics and Genomics Programme, Centre for Genomic Regulation, 08003 Barcelona, Spain; § Departament de Ciències Experimentals I de la Salut, Universitat Pompeu Fabra, 08003 Barcelona, Spain; ** Institució Catalana de Recerca i Estudis Avançats, 08010 Barcelona, Spain

**Keywords:** gentisate pathway, 3-oxoadipate pathway, catabolism of hydroxybenzoates, mitochondrial carrier, evolution of biochemical pathways

## Abstract

The pathogenic yeast *Candida parapsilosis* metabolizes hydroxyderivatives of benzene and benzoic acid to compounds channeled into central metabolism, including the mitochondrially localized tricarboxylic acid cycle, via the 3-oxoadipate and gentisate pathways. The orchestration of both catabolic pathways with mitochondrial metabolism as well as their evolutionary origin is not fully understood. Our results show that the enzymes involved in these two pathways operate in the cytoplasm with the exception of the mitochondrially targeted 3-oxoadipate CoA-transferase (Osc1p) and 3-oxoadipyl-CoA thiolase (Oct1p) catalyzing the last two reactions of the 3-oxoadipate pathway. The cellular localization of the enzymes indicates that degradation of hydroxyaromatic compounds requires a shuttling of intermediates, cofactors, and products of the corresponding biochemical reactions between cytosol and mitochondria. Indeed, we found that yeast cells assimilating hydroxybenzoates increase the expression of genes *SFC1*, *LEU5*, *YHM2*, and *MPC1* coding for succinate/fumarate carrier, coenzyme A carrier, oxoglutarate/citrate carrier, and the subunit of pyruvate carrier, respectively. A phylogenetic analysis uncovered distinct evolutionary trajectories for sparsely distributed gene clusters coding for enzymes of both pathways. Whereas the 3-oxoadipate pathway appears to have evolved by vertical descent combined with multiple losses, the gentisate pathway shows a striking pattern suggestive of horizontal gene transfer to the evolutionarily distant Mucorales.

Enzymatic degradation of lignin to simpler aromatic compounds containing substituted benzene ring is accomplished by soil bacteria and fungi (Kirk and Farrell 1987; Bugg *et al.* 2011). Yeast species from the “CTG” clade of Saccharomycotina can utilize a range of hydroxyderivatives of benzene and benzoic acid as the sole carbon source. In the pathogenic yeast *Candida parapsilosis*, these compounds are metabolized via the hydroxyhydroquinone (HHQ) variant of the 3-oxoadipate pathway (3-OAP) and the glutathione-dependent variant of the gentisate pathway (GP) (Middelhoven *et al.* 1992; Middelhoven 1993). Previously, we have identified *C. parapsilosis* genes *MNX1*, *MNX2*, *MNX3*, *GDX1*, *HDX1*, and *FPH1* coding for components of both pathways (Holesova *et al.* 2011). We have found that the genes coding for key enzymes of the GP and 3-OAP are organized in two metabolic clusters (Holesova *et al.* 2011; Gerecova *et al.* 2015). These genes exhibit very low level of expression in cells cultivated in synthetic media containing glucose. However, they are highly induced in cells assimilating hydroxyaromatic compounds such as 4-hydroxybenzoate (4OH), hydroquinone, resorcinol (in 3-OAP), 3-hydroxybenzoate (3OH), or gentisate (in GP).

The final products of the 3-OAP are succinate and acetyl-coenzyme A (acetyl-CoA), while the GP yields fumarate and pyruvate. These metabolites are substrates for pyruvate dehydrogenase complex, tricarboxylic acid (TCA) cycle, and other reactions leading to synthesis of heme, amino acids, and fatty acids (Hoffman *et al.* 2003; Tehlivets *et al.* 2007; Han *et al.* 2011; Herzig *et al.* 2012). This suggests that mitochondria play an important role in the integration of both pathways with the central metabolism. However, it remains unknown how this is orchestrated. Specific mitochondrial carriers (MCs) can transport the products or intermediates of hydroxybenzoate catabolism across the inner mitochondrial membrane, thus interconnecting biochemical reactions operating in the cytosol and mitochondria. In general, MCs ensure transport of substrates, such as nucleotides, amino acids, cofactors, carboxylic acids, and inorganic ions required for oxidative phosphorylation, gluconeogenesis, synthesis and degradation of amino acids and fatty acids (Palmieri and Pierri 2010). Absence of individual MC can impair or prevent the growth of yeast cells on nonfermentable carbon sources, cause instability of mitochondrial DNA (mtDNA), or perturb other cellular functions depending on the transported substrates (Trézéguet *et al.* 1999; Palmieri *et al.* 2006).

The aim of this study was to investigate subcellular localization of enzymes involved in the 3-OAP and GP, and the expression of corresponding genes in *C. parapsilosis* cells utilizing 3OH and 4OH. We examined the expression of MCs potentially involved in the transport of metabolites generated in the catabolism of hydroxybenzoates. Furthermore, we investigated the evolution of both pathways and their connection with mitochondrial metabolism via MCs. Our results revealed that genes encoding orthologs of succinate/fumarate carrier (Sfc1p), coenzyme A (CoA) carrier (Leu5p), oxoglutarate/citrate carrier (Yhm2p), and the subunit of pyruvate carrier (Mpc1p) are induced in cells utilizing hydroxybenzoates, and seem to provide a key functional link between the catabolism of hydroxyaromatic compounds and central metabolic pathways. Finally, we used phylogenetic profiling to trace the evolution of both pathways, with the dual objective of potentially discovering putative new components and elucidating the origin of the patchy phylogenetic distribution of these pathways. Our results demonstrate distinct evolutionary trajectories for the two metabolic gene clusters. The 3-OAP appears to have evolved by vertical descent combined with multiple losses, resulting in its current sparse distribution. In contrast, the GP shows a striking pattern suggestive of horizontal gene transfer to the evolutionarily distant Mucorales, which share a similar organization of the gene cluster.

## Materials and Methods

### Microbial strains and cultivation conditions

*C. parapsilosis* strains CLIB214 (identical to CBS604^T^), CDU1 (CLIB 214 *ura3*Δ::*FRT*/*ura3*Δ::*FRT*) (Ding and Butler 2007), SR23 (*ade*^-^, *lys4*^-^) (Nosek *et al.* 2002) and *Saccharomyces cerevisiae* strains BY4742 (MATα, *his3-1*, *leu2-0*, *lys2-0*, *ura3-0*) and BY4742 Δ*sfc1* (BY4742 *sfc1*::*kanMX*) (Euroscarf) were used in this study. Yeasts were cultivated in synthetic media [0.67% (w/v) yeast nitrogen base w/o amino acids (Difco)] containing 2% (w/v) glucose (SD); 10 mM 3-hydroxybenzoate (S3OH); 10 mM 4-hydroxybenzoate (S4OH); 3% (w/v) glycerol (SGly); 3% (w/v) glycerol + 2% (w/v) galactose (SGlyGal_2.0_) or 3% (w/v) glycerol + 0.1% (w/v) galactose (SGlyGal_0.1_). The media were supplemented with appropriate amino acids and bases according to the strain requirements. Hydroxyaromatic compounds were dissolved in dimethyl sulfoxide (DMSO) as 0.5 M stocks. Agar (2% w/v) was added for solid media. *Escherichia coli* strain DH5α [F^−^, *φ80dlacZ*Δ*M15*, Δ*(lacZYA-argF) U169*, *deoR*, *recA1*, *endA1*, *hsdR17 (rk*^-^, *mk^+^)*, *λ*, *thi-1*, *gyrA96*, *relA1*, *glnV44*, *nupG*] (Life Technologies) was used in cloning experiments and plasmid propagation.

### Preparation of plasmid constructs

Coding sequences of *C. parapsilosis* genes *FPH1*, *HDX1*, *OSC1*, *OCT1*, and *SFC1* were amplified by polymerase chain reaction (PCR) using genomic DNA of the strain CBS604^T^ and gene specific primers listed in Supplemental Material, Table S1. The PCR products were cloned into pDrive vector (Qiagen). The pDrive-derived constructs containing *HDX1*, *OSC1*, *OCT1*, or *FPH1* genes were digested with *Xba*I endonuclease, and DNA fragments with corresponding genes were inserted into the *Xba*I site of the pBP8 vector (Kosa *et al.* 2007). Resulting plasmids were named pBP8-*HDX1*, pBP8-*OSC1*, pBP8-*OCT1*, and pBP8-*FPH1*. Next, the pDrive-*SFC1* construct was digested with *Not*I endonuclease, and the DNA fragment containing the coding sequence of *SFC1* was inserted into the *Not*I site of the pYES2/CT vector (Life Technologies) generating the plasmid pYES2/CT-*SFC1*. To construct the plasmid pUG36-*SFC1*, the *Hin*dIII–*Ava*I fragment of pYES2/CT-*SFC1* was inserted into corresponding sites of the pUG36 vector (provided by J. H. Hegemann, Heinrich-Heine-Universität, Düsseldorf, Germany). To prepare construct pPK6-*SFC1*, the *SFC1* coding sequence was amplified by PCR using specific primers (Table S1) on the pYES2/CT-*SFC1* template and inserted into the *Sma*I site of the pPK6 vector (Kosa *et al.* 2007). The plasmid constructs used in this study are listed in Table S2. Plasmid DNAs were introduced into yeast cells by electroporation (Zemanova *et al.* 2004), or using the lithium/polyethylene glycol/carrier DNA protocol (Gietz *et al.* 1995).

### RNA-seq analysis

Total RNA was isolated from cells grown in SD, S3OH, and S4OH media according to the protocol using phenol-chloroform extraction described by Cross and Tinkelenberg (1991). RNA was treated with RNase-free DNase I (New England Biolabs), extracted with phenol:chloroform (1:1) and purified using a clean up protocol of the GeneJET RNA purification kit (Thermo Scientific). The quality of RNA was assessed with RNA nanochip on Agilent Bioanalyzer 2100. RNA samples with RNA integrity number (RIN) above 7.0 were sequenced by Illumina GAIIx platform using 2 × 50 pair-end reads. Reads were aligned on the *C. parapsilosis* reference genome using tophat2 v2.0.6 (Kim *et al.* 2013) with default parameters. Differentially expressed genes were detected using cufflinks v2.1.1 (Trapnell *et al.* 2013) using a *P*-value cut-off of 0.05, and fold change > 2. Statistical analyses were conducted using cummeRbund package.

### Quantitative PCR analysis of mRNA

Total RNA was isolated from *C. parapsilosis* CLIB214 either by extraction with hot acid phenol (Collart and Oliviero 2001) or phenol-chloroform (Cross and Tinkelenberg 1991). RNA preparations were treated with RNase-free DNase I (New England Biolabs) and cDNA was synthesized using Maxima H Minus First Strand cDNA Synthesis Kit with oligo(dT) primers (Thermo Scientific) in a total volume of 20 μl. Quantitative real-time PCR (RT-qPCR) was performed on cDNA template using gene-specific primers (Table S1) and Luminaris Color HiGreen High ROX qPCR Master Mix (Thermo Scientific) in a StepOne cycler (Applied Biosystems). All reactions were carried out in technical duplicates, and normalized to *EFB1* (translation elongation factor EF-1 beta) mRNA. Relative mRNA levels for all genes were determined in at least three independent experiments and calculated by the relative standard curve method according to the manufacturer’s instructions (Applied Biosystems). The significance of differences between the samples and the control (SD medium) was evaluated by Student’s *t*-test and was considered significant when *P* < 0.05.

### Functional complementation of the S. cerevisiae Δsfc1 mutant

Plasmids pYES2/CT and pYES2/CT-*SFC1* were transformed into the *S. cerevisiae* strains BY4742 and BY4742 Δ*sfc1*. The transformants were then grown in SD medium to midexponential phase, diluted to OD_600_ = 0.5, and 5 μl of serial fivefold dilutions were spotted on solid SGlyGal_0.1_ medium. Growth was evaluated either after a 2 d incubation at 28° or after 7 d at 20 and 37°.

### Fluorescence microscopy

Intracellular localization of proteins Hdx1, Osc1, Oct1, and Fph1 C-terminally tagged with yEGFP3 was observed in *C. parapsilosis* CDU1 cells by fluorescence microscopy. Localization of *C. parapsilosis* Sfc1p N-terminally tagged with yEGFP3 was analyzed in *S. cerevisiae* strains BY4742 and BY4742 Δ*sfc1*, and in *C. parapsilosis* strain SR23. Yeast cells transformed with plasmid constructs were grown at 28° in synthetic media containing appropriate carbon sources and midexponential phase cells were visualized using a BX50 microscope equipped with the appropriate filter set and digital camera DP70 (Olympus Optical). DNA in living cells was stained with 4′,6-diamidino-2-phenylindole (DAPI) at a concentration of 0.5 µg/ml for 1 hr at 28°. Mitochondria were stained with 25 nM MitoTracker Red CMXRos (Molecular Probes) for 15–60 min at room temperature.

### Bioinformatics analyses

The following databases were used to identify gene and protein sequences: *Candida* Genome Database (http://www.candidagenome.org) and *Saccharomyces* Genome Database (http://www.yeastgenome.org). Geneious 5.6.6 software from Biomatters (Kearse *et al.* 2012) was used to align protein sequences using MAFFT program v7.017 (Katoh *et al.* 2002), predict transmembrane segments (Transmembrane prediction tool 0.9 plugin), and construct a phylogenetic tree of MCF proteins (PhyML plugin v. 2.1.0). The orthologous genes were identified using the *Candida* Gene Order Browser (http://cgob3.ucd.ie/; Fitzpatrick *et al.* 2010; Maguire *et al.* 2013).

### Phylogenetic profiling

The phylogenetic profiles of *C. parapsilosis* proteins were generated by blasting them against 69 Saccharomycetes, 10 *Aspergillus*, and six *Fusarium* proteomes available in MetaPhOrs database as of June 2014 (Pryszcz *et al.* 2011). We considered the presence of a homolog of the *C. parapsilosis* protein in a given species if it had a Blast hit below a stringent threshold of (*E*-value <10^−25^), otherwise the protein was considered to be absent. Proteins with a similar phylogenetic profile as those identified in the cluster were detected by computing the Hamming distance (Gabaldón 2008) to the phylogenetic profiles of Hdx1p (3-OAP) or Gdx1p (GP).

Phylogenetic histories of the relevant genes were first inspected using the Maximum Likelihood phylogenies available in PhylomeDB (Huerta-Cepas *et al.* 2014), and reconstructing phylogenies of these proteins, and their closest 350 blast hits, in the NCBI nonredundant database searched as of January 2016. In brief, phylogenies were reconstructed as follows: protein sequences of the hits that passed a threshold of similarity (*E*-value <10^−5^) and coverage (>33% aligned over the query sequence), were aligned with MUSCLE (Edgar 2004) with default parameters, trimmed with trimAl v1.4 (Capella-Gutiérrez *et al.* 2009) to eliminate alignment columns with > 50% gaps. A Maximum Likelihood phylogenetic reconstruction was performed with PhyML v3 (Guindon and Gascuel 2003) using the LG model, approximating four rate categories and the fraction of invariable sites from the data. The chromosomal locations of the *C. parapsilosis* genes, and their homological Mucorales genes, were inspected using the *Candida* Gene Order Browser (Maguire *et al.* 2013), or the *Phycomyces* and *Mucor* Gene Order Browser (Corrochano *et al.* 2016).

### Data availability

Yeast strains and plasmid constructs are available upon request. Sequencing reads of the RNA-seq experiment have been deposited to the European Nucleotide Archive—Short Read Archive (ENA-SRA; http://www.ebi.ac.uk/ena/data/view/PRJEB1707).

## Results and Discussion

### Identification of the genes coding for 3-oxoadipate CoA-transferase and 3-oxoadipyl-CoA thiolase

**Figure 1:**
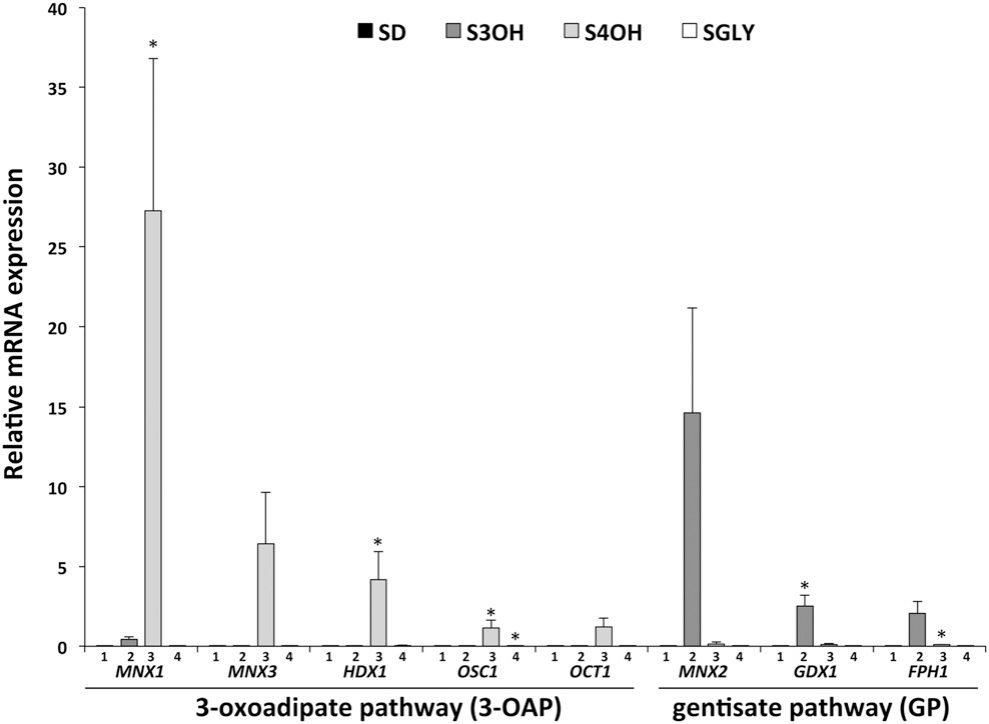
Relative mRNA expression of *C. parapsilosis* genes involved in the 3-OAP (*MNX1*, *MNX3*, *HDX1*, *OSC1*, and *OCT1*) and GP (*MNX2*, *GDX1*, and *FPH1*). RNA samples were prepared from CLIB214 cells grown in synthetic medium supplemented with 2% glucose (SD, bars no. 1), 10 mM 3-hydroxybenzoate (S3OH, bars no. 2), 10 mM 4-hydroxybenzoate (S4OH, bars no. 3), or 3% glycerol (SGly, bars no. 4). Quantification of mRNA was performed as described in *Materials and Methods*. Relative expression was normalized to *EFB1* gene expression. The assays were performed in at least three independent experiments with two parallel replicates in each case (error bars, mean ± SEM) and the significance of differences between the samples (S3OH, S4OH, and SGly) and the control (SD) was evaluated by Student’s *t*-test (* *P* < 0.05).

Previously, we have identified several *C. parapsilosis* genes coding for key enzymes of the 3-OAP (*MNX1*, *MNX3*, and *HDX1*) and GP (*MNX2*, *GDX1*, and *FPH1*) (Holesova *et al.* 2011). In this study, we searched for genes coding for additional components of these pathways using the amino acid sequences of bacterial enzymes as queries in BlastP searches of the *C. parapsilosis* genome sequence. The queries derived from the sequences of two 3-oxoadipate CoA-transferase subunits from *Pseudomonas putida* (PcaI and PcaJ) revealed a single ORF CPAR2_406450 (*E*-value <10^−45^). The deduced amino acid sequence contains two domains typical for CoA-transferase family I enzymes, and a predicted N-terminal mitochondrial import sequence (21-aa; MitoProt II score 0.8559). This indicates that corresponding enzymatic reaction (*i.e.*, the conversion of 3-oxoadipate to 3-oxoadipyl-CoA) occurs inside of mitochondria. Moreover, our results of RNA-seq and RT-qPCR analyses (see below) demonstrate that this gene is selectively upregulated in cells assimilating 4OH, a substrate of the 3-OAP (Figure 1 and Table S3). The ORF is located downstream from the gene *HDX1* within the gene cluster, which is conserved in other yeast species (*e.g.*, *C. albicans*, *C. dubliniensis*, *C. orthopsilosis*, *C. tropicalis*, *Debaryomyces hansenii*, and *Scheffersomyces stipitis*) possessing the 3-OAP. These data support the role of the CPAR2_406450 product in this pathway and therefore, we named this gene *OSC1* for 3-oxoadipate:succinyl-CoA transferase 1.

Next, we searched for a homolog of 3-oxoadipyl-CoA thiolase, which catalyzes the last reaction of the 3-OAP. We performed BlastP analysis using the sequence of PcaF protein from *P. putida*. This analysis revealed four ORFs (*E*-value <10^−56^) CPAR2_212970, CPAR2_212810, CPAR2_503690, and CPAR2_800020 coding for proteins with predicted N- and C-terminal thiolase domains. These ORFs are orthologs of *C. albicans POT1-2*, *ERG10*, *FOX3*, and *POT1* genes, respectively (Otzen *et al.* 2013). While *ERG10* codes for acetyl-CoA acetyltransferase, which has a role in ergosterol biosynthesis, Fox3p and Pot1p are presumed peroxisomal 3-oxoacyl-CoA thiolases. Although Pot1-2p is annotated as another putative peroxisomal 3-oxoacyl-CoA thiolase, it has typical N-terminal sequence for import into mitochondria (38-aa; MitoProt II score 0.9651). Our RNA-seq data indicate that the transcription of CPAR2_212970, CPAR2_503690, and CPAR2_800020 is induced on media with hydroxyaromatic substrate (Table S3). However, only CPAR2_212970 (like *MNX1*, *MNX3*, *HDX1*, and *OSC1*) appears to be selectively upregulated in cells utilizing 4OH, a substrate of the 3-OAP, but not 3OH, which is metabolized via the GP (Table S3). Moreover, similar to its ortholog from *C. albicans* (Pot1-2p), CPAR2_212970 has typical N-terminal sequence for import into mitochondria (17-aa; MitoProt II score 0.9296). These results indicate that CPAR2_212970 encodes 3-oxoadipyl-CoA thiolase, catalyzing the conversion of 3-oxoadipyl-CoA to final products of the 3-OAP (*i.e.*, acetyl-CoA and succinate). This is in line with the prediction that also 3-oxoadipate CoA-transferase (Osc1p), generating the substrate for 3-oxoadipyl-CoA thiolase, is imported into mitochondria, and we named the gene CPAR2_212970 as *OCT1* for 3-oxoadipyl-CoA thiolase 1.

In contrast to Osc1p and Oct1p, the BlastP searches using the sequences of maleylacetate reductase (MacA) and maleylpyruvate isomerase (Mpi) from *P. putida* did not reveal any clear candidates for these enzymes in *C. parapsilosis*.

### The 3-OAP and GP genes are highly induced in cells assimilating hydroxybenzoates

RNA-seq analysis of the transcriptome in *C. parapsilosis* cells grown in synthetic media with 3OH, 4OH, or glucose as the sole carbon source was used to assess the expression of genes involved in the 3-OAP and GP (Table S3, Table S4, and Table S5). Relative mRNA expression of the 3-OAP genes is very low in glucose medium. The induction of individual genes in S4OH medium was 3541-fold (*MNX1*), 476-fold (*MNX3*), 255-fold (*HDX1*), 331-fold (*OSC1*), and 67-fold (*OCT1*). The genes encoding the GP enzymes are highly upregulated in cells grown in S3OH medium reaching 1109-fold (*MNX2*), 344-fold (*GDX1*), and 245-fold (*FPH1*) increase compared to SD medium.

To validate RNA-seq data, we investigated expression of these genes by RT-qPCR analysis. This experiment confirmed very low levels of expression in glucose medium, and the specific induction by the corresponding hydroxybenzoate (Figure 1 and Table S6). These results are also in line with the activities of *MNX1*, *MNX2*, *MNX3*, and *GDX1* promoters fused with *β*-galactosidase described previously (Holesova *et al.* 2011). The genes *MNX1* and *MNX2* encoding the first enzyme in the 3-OAP and GP, respectively, exhibit the highest induction of expression in cells grown on hydroxybenzoate compared to SD medium. Namely, the expression of *MNX1* is induced 2701-fold on S4OH, and expression of *MNX2* is induced 3271-fold on S3OH. The genes from both pathways (*MNX1*, *MNX2*, *GDX1*, and *FPH1*) are only weakly induced in media with a hydroxyaromatic compound not degraded by the particular pathway (*e.g.*, *MNX1* is induced by 42-fold on S3OH, and *MNX2* 33-fold on S4OH). However, this corresponds to only 1–5% of the highest induction achieved in media containing the catabolized hydroxybenzoate. The relative expression of these genes is slightly higher in cells grown in medium containing glycerol as a respiratory substrate (SGly) than in SD medium (1.3- to 3.2-fold; Table S6), but the differences are not statistically significant when compared with the expression induced by hydroxybenzoates (Figure 1).

### Intracellular localization of the 3-OAP and GP enzymes

**Figure 2:**
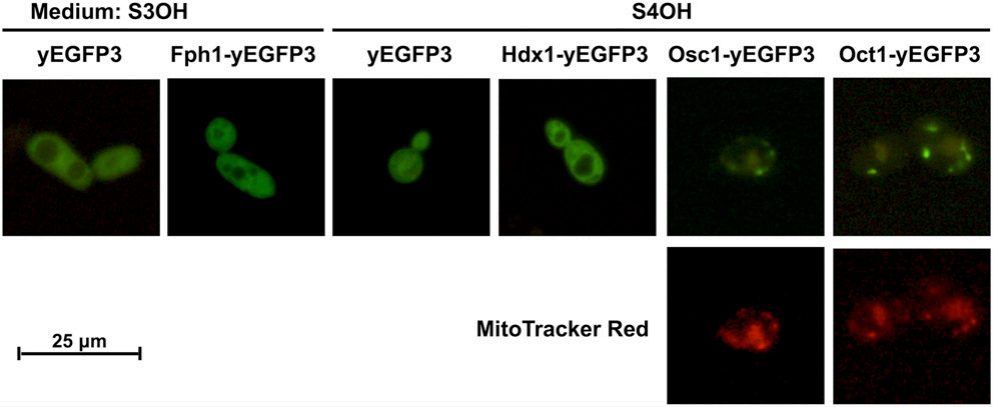
Intracellular localization of Hdx1p, Osc1p, Oct1p involved in the 3-OAP and Fph1p involved in the GP. *C. parapsilosis* strain CDU1 was transformed with pBP8-derived plasmid constructs expressing indicated proteins fused with yEGFP3. Transformants with the vector pBP8 were used as a control. Cells were grown at 28° in synthetic media containing 3-hydroxybenzoate (GP), or 4-hydroxybenzoate (3-OAP), as a sole carbon source, and examined by fluorescence microscopy. Mitochondria were stained with MitoTracker Red CMXRos as described in *Materials and Methods*. Bar, 25 μm.

Degradation of hydroxyaromatic compounds in *C. parapsilosis* cells could proceed in the cytosol and/or in specialized subcellular compartments such as mitochondria or peroxisomes. The final products of the GP (fumarate and pyruvate) and 3-OAP (succinate and acetyl-CoA) are molecules that could be at least partially metabolized in mitochondria, *e.g.*, in the TCA cycle. Previously, we investigated subcellular localization of several 3-OAP (Mnx1p, Mnx3p) and GP (Mnx2p, Gdx1p) enzymes catalyzing first two steps of the corresponding metabolic pathways (Holesova *et al.* 2011). Our study revealed that the fusion proteins Mnx1-yEGFP3 and Mnx3-yEGFP3 localize in the cytoplasm, and Mnx2-yEGFP3 and Gdx1-yEGFP3 associate with mitochondria. As both Mnx2p and Gdx1p lack a mitochondrial import presequence, it has been speculated that these proteins can associate with the outer mitochondrial membrane. Hence, the reactions catalyzed by all four enzymes occur in the cytoplasm. Here, we examined localization of additional enzymes involved in both pathways. First, we predicted protein import into mitochondria using the MitoProt II tool (Table S7) and, subsequently, we analyzed subcellular localization of Hdx1, Osc1, Oct1, and Fph1 proteins tagged with yEGFP3 in *C. parapsilosis* cells cultivated in synthetic media with 4OH and 3OH, which induce the 3-OAP and GP, respectively (Figure 2).

The sequence of Hdx1p lacks a putative N-terminal signal sequence for import into mitochondria, and we observed the Hdx1-yEGFP3 protein in the cytoplasm (Figure 2). On the other hand, Osc1p and Oct1p contain a typical N-terminal mitochondrial presequence (MitoProt II score 0.8559 and 0.9296, respectively), and we detected Osc1-yEGFP3 and Oct1-yEGFP3 proteins as discrete spots in cells grown in S4OH medium. Costaining with MitoTracker Red confirmed mitochondrial localization of both fusion proteins. These results indicate that the last two steps of the 3-OAP occur in mitochondria, and suggest that intermediates of the 3-OAP pathway, such as 2-maleylacetate or 3-oxoadipate, are likely transported into mitochondria by specific MC proteins. In the case of Fph1p, catalyzing the final step of the GP, we identified a putative N-terminal mitochondrial import signal (MitoProt II score 0.8244). However, the Fph1p does not seem to be imported into mitochondria, as the Fph1-yEGFP3 appears to be distributed throughout the cytoplasm similarly to the control yEGFP3 protein (Figure 2). This indicates that the reactions of the GP occur in the cytoplasm, and the final products of this pathway (fumarate and pyruvate) are transported into mitochondria via succinate/fumarate (Sfc1p) and pyruvate (Mpc1p/Mpc3p) carriers, respectively.

### Inventory of mitochondrial carriers in C. parapsilosis

To initiate the investigation of MCs that could be implicated in the transport of metabolites of the 3-OAP and GP, we compiled a list of putative mitochondrial carrier family (MCF) proteins in *C. parapsilosis* using data available in the *Candida* Genome Database (Table S8). The genes encoding proteins with domains inherent for MCs (PFAM00153, INTERPRO IPR001993 and PROSITE PS50920) containing highly conserved signature motif P-X-[D/E]-X-X-[K/R] were selected. There are 33 genes coding for MCF proteins in *C. parapsilosis*, of which 32 are orthologs of *S. cerevisiae* MCs. *S. cerevisiae* does not contain a homolog of CPAR2_502880 protein, which has unknown function. The *C. parapsilosis* MCF proteins can be classified into four major groups (Figure S1). Affiliation of particular carriers to a specific group could be useful for designing an experimental strategy that will identify transported substrates, as only the ADP/ATP carrier encoded by the *AAC1* gene has been functionally characterized so far in *C. parapsilosis* (Nebohacova *et al.* 1999). The list of MCs includes also two orthologs of the subunits of pyruvate carrier, Mpc1p and Mpc3p (Bricker *et al.* 2012; Herzig *et al.* 2012), although they do not belong to the MCF. In contrast to *S. cerevisiae*, the *C. parapsilosis* genome does not encode an ortholog of *Sc*Mpc2p, which is interchangeable with *Sc*Mpc3p.

### Expression of the genes encoding mitochondrial carriers

RNA-seq analysis revealed relative levels of transcripts in *C. parapsilosis* cells grown in media containing 3OH, 4OH, or glucose as the sole carbon source. Among the genes coding for MCs, *AAC1* and *MIR1* (encoding phosphate carrier) exhibit the highest level of expression on all three substrates, although they do not appear to be significantly upregulated by hydroxyaromatic compounds (Table S3). These MCs import ADP and P_i_ into mitochondria, where they serve primarily as substrates for ATP synthase. In contrast, *SFC1* is the most induced MC gene (15-fold increase in expression) in media with either 3OH or 4OH when compared with the expression in SD medium (Table S3).

To verify and quantify expression data obtained by RNA-seq experiment, we performed RT-qPCR analysis in *C. parapsilosis* cells, assimilating 3OH or 4OH as the sole carbon source. Control growth substrates were glucose and glycerol. We analyzed 11 genes for MCs presumably important for metabolism of cells utilizing 3OH or 4OH, as they could transport final products, intermediates, or cofactors of the GP and 3-OAP. The list includes MCs potentially transporting keto acids, as the GP and 3-OAP contain molecules with carboxyl and/or keto groups. Their orthologs in *S. cerevisiae* were functionally characterized. Sfc1p is a succinate/fumarate carrier (Palmieri *et al.* 1997), and Odc1p is an oxodicarboxylate carrier transporting 2-oxoadipate, 2-oxoglutarate, and other C_5_–C_7_ (oxo)dicarboxylates with high affinity (Palmieri *et al.* 2001). Dic1p is a dicarboxylate carrier importing malate, succinate, and malonate into mitochondria in exchange for phosphate (Palmieri *et al.* 1996; Kakhniashvili *et al.* 1997). Yhm2p is an oxoglutarate/citrate antiporter that can transport also succinate, fumarate and oxaloacetate (Castegna *et al.* 2010). Oac1p is transporting oxaloacetate, (thio)sulfate, malonate, and isopropylmalate (Palmieri *et al.* 1999b; Marobbio *et al.* 2008). It was suggested that Ymc1p and Ymc2p overlap in transport functions with Odc1p and Odc2p in *S. cerevisiae* (Trotter *et al.* 2005). Leu5p is necessary for the accumulation of CoA in the mitochondrial matrix (Prohl *et al.* 2001). Crc1p mediates carnitine-dependent transport of acetyl-CoA into mitochondria (Palmieri *et al.* 1999a; van Roermund *et al.* 1999). Mpc1p and Mpc3p are subunits of carrier importing pyruvate into mitochondria (Bricker *et al.* 2012; Herzig *et al.* 2012).

**Figure 3:**
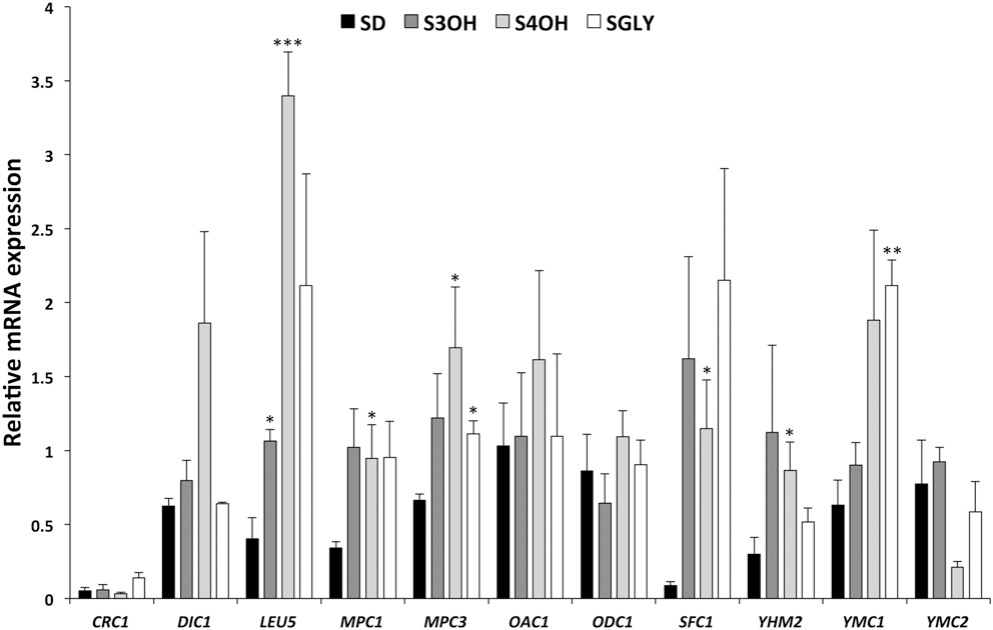
Relative mRNA expression of the *C. parapsilosis* MC genes (*CRC1*, *DIC1*, *LEU5*, *MPC1*, *MPC3*, *OAC1*, *ODC1*, *SFC1*, *YHM2*, *YMC1*, and *YMC2*). RNA samples were prepared from CLIB214 cells grown in synthetic medium supplemented with 2% glucose (SD), 10 mM 3-hydroxybenzoate (S3OH), 10 mM 4-hydroxybenzoate (S4OH), or 3% glycerol (SGly). Quantification of mRNA was performed as described in *Materials and Methods*. Relative expression was normalized to *EFB1* gene expression. The assays were performed in at least three independent experiments, with two parallel replicates in each case (error bars, mean ± SEM), and the significance of differences between the samples (S3OH, S4OH, and SGly) and the control (SD) was evaluated by Student’s *t*-test (* *P* < 0.05, ** *P* < 0.01, *** *P* < 0.001).

Our results show that *SFC1* is the most induced MC gene in *C. parapsilosis*, both in S3OH and S4OH media, with fold change of 18.5 and 13.1, respectively (Figure 3). The expression of *SFC1*, *LEU5*, *YHM2*, and *MPC1* genes was at least two times higher in both hydroxybenzoate-containing media. The expression of *DIC1*, *YMC1*, and *MPC3* genes was induced at least twofold only in S4OH medium. The expression of *ODC1* and *OAC1* is relatively high under all growth conditions. However, not all MC genes are induced by glycerol, suggesting that transport function of some MCs is needed for the cells grown on all substrates including glucose. The genes *SFC1* and *LEU5* are induced at least fivefold, *YMC1*, *MPC1*, and *CRC1* at least twofold. The expression of genes *YHM2* and *MPC3* is increased 1.7-fold by glycerol. Transcription analysis of several corresponding carriers in *S. cerevisiae* showed that respiratory substrates induce the expression of *SFC1* (Fernandez *et al.* 1994), *YMC1* (Trotter *et al.* 2005), *CRC1*, *MPC3*, but not *MPC1* (Timon-Gomez *et al.* 2013) and *ODC2* (Palmieri *et al.* 2001).

### C. parapsilosis SFC1 functionally complements Δsfc1 in S. cerevisiae

It is evident from the gene expression analysis that *SFC1* (CPAR2_100870) has an important role in the metabolism of hydroxybenzoates. To verify that it encodes the functional succinate/fumarate carrier, we examined this gene in more detail. The deduced sequence of the Sfc1 protein shares 66% identity (76% similarity) with its ortholog from *S. cerevisiae* (*Sc*Sfc1p/YJR095W). Sequence comparison of succinate/fumarate carriers (Figure S2) shows three “mitochondrial energy transfer signature” motifs with conserved amino acids. These motifs are furthermore identical with sequences in the well-characterized succinate/fumarate carrier from *S. cerevisiae*. Seven amino acids constituting contact points for substrate binding in the *Sc*Sfc1p (Kunji and Robinson 2006) are conserved in *C. parapsilosis* Sfc1p. This further supports the hypothesis that this protein is a functional succinate/fumarate carrier in *C. parapsilosis*.

**Figure 4:**
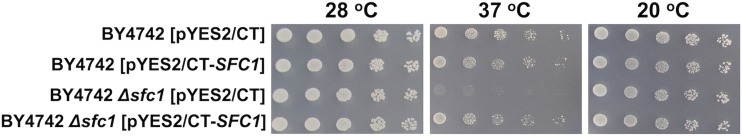
Functional complementation of *S. cerevisiae* Δ*sfc1* by the *SFC1* gene from *C. parapsilosis*. Strains BY4742 and BY4742 Δ*sfc1* transformed with pYES2/CT (control) and pYES2/CT-*SFC1* were spotted as serial dilutions onto SGlyGal_0.1_ plates. Growth was evaluated either after 2 d incubation at 28° (left panel) or after 7 d at 37° (middle panel) and 20° (right panel).

To test this idea experimentally, we expressed the *C. parapsilosis SFC1* gene in *S. cerevisiae* cells. Our approach was essentially the same as that described previously for the *AAC1* gene encoding ADP/ATP carrier (Nebohacova *et al.* 1999). Comparison of *S. cerevisiae* strains BY4742 and BY4742 Δ*sfc1* at normal, suboptimal, and elevated temperature revealed that the mutant strain does not grow at 37° in media with 3% glycerol (Figure 4). Next, the *C. parapsilosis SFC1* gene was expressed from the multicopy plasmid pYES2/CT-*SFC1* under control of the *GAL1* promoter induced by 0.1% galactose to evaluate its function *in vivo*. Heterologous expression restored the ability of BY4742 Δ*sfc1* strain to grow on media with respiratory substrate at 37°, implying that an obligatory function performed by *Sc*Sfc1p to support growth was compensated by the activity of its *C. parapsilosis* homolog. The expression of the *C. parapsilosis SFC1* gene had no visible effect on the growth of wild-type strain BY4742.

**Figure 5:**
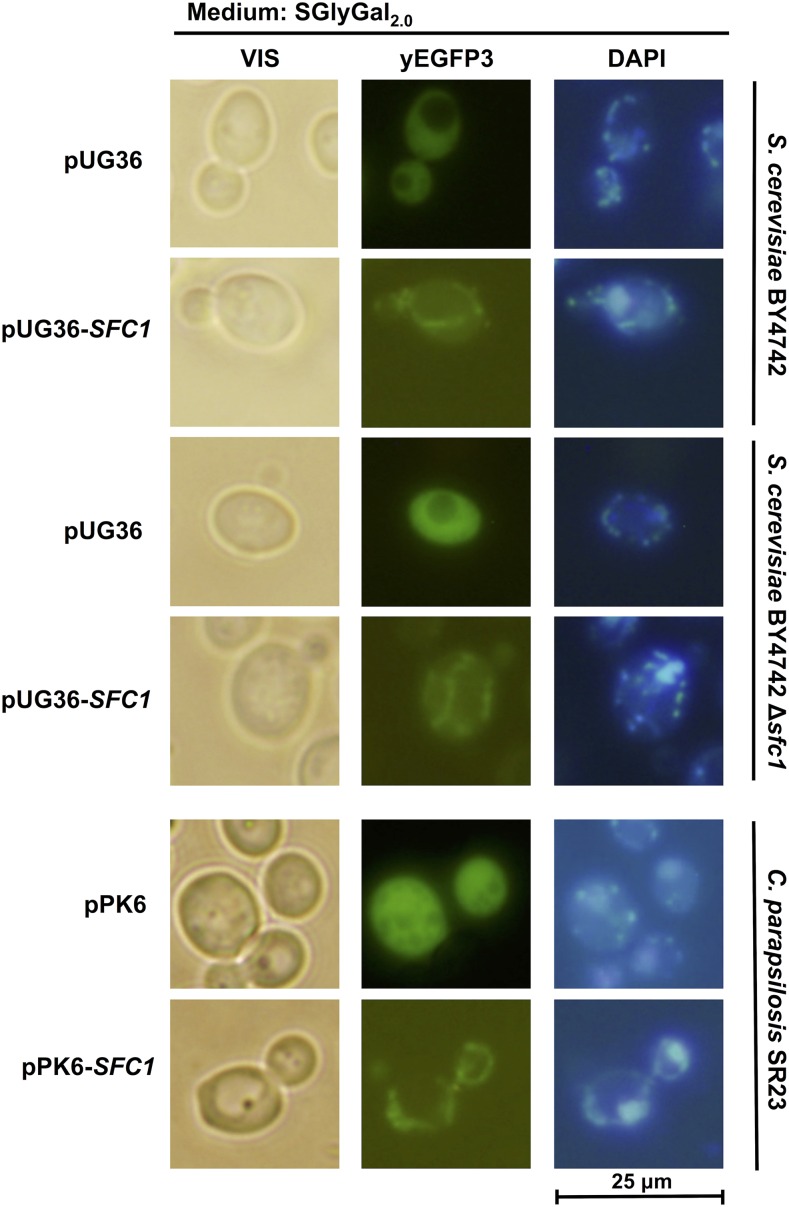
Intracellular localization of *C. parapsilosis* Sfc1p. *S. cerevisiae* strains BY4742 and BY4742 Δ*sfc1* were transformed with plasmid pUG36-*SFC1*. *C. parapsilosis* strain SR23 was transformed with plasmid pPK6-*SFC1*. Cells transformed with the vector pUG36 (*S. cerevisiae*) and pPK6 (*C. parapsilosis*) were used as controls. Transformants were grown at 28° in SGlyGal_2.0_ medium and examined by fluorescence microscopy. DNA in cells was stained with DAPI as described in *Materials and Methods*. Bar, 25 μm.

Next, we examined the subcellular localization of *C. parapsilosis* Sfc1p using the plasmid pUG36-*SFC1*, which allows the expression of Sfc1p tagged at its N-terminus with yEGFP3. Figure 5 shows intracellular localization of Sfc1p in *S. cerevisiae* cells, in both the wild-type strain and the Δ*sfc1* mutant. Staining of cells with DAPI demonstrated mitochondrial localization of Sfc1p, with a partial overlap with mitochondrial nucleoids. Similar results were obtained when yEGFP3-Sfc1p was expressed in *C. parapsilosis* from the plasmid pPK6-*SFC1* (Figure 5). Most of the green fluorescence signal was focused in the subcellular compartments containing mtDNA, as demonstrated by staining with DAPI. This is in line with the presence of Sfc1p in the fraction of mitochondrial nucleoids isolated from *C. parapsilosis* cells (Miyakawa *et al.* 2009).

### Interconnection of cytosolic and mitochondrial metabolism of hydroxyaromatic substrates

**Figure 6:**
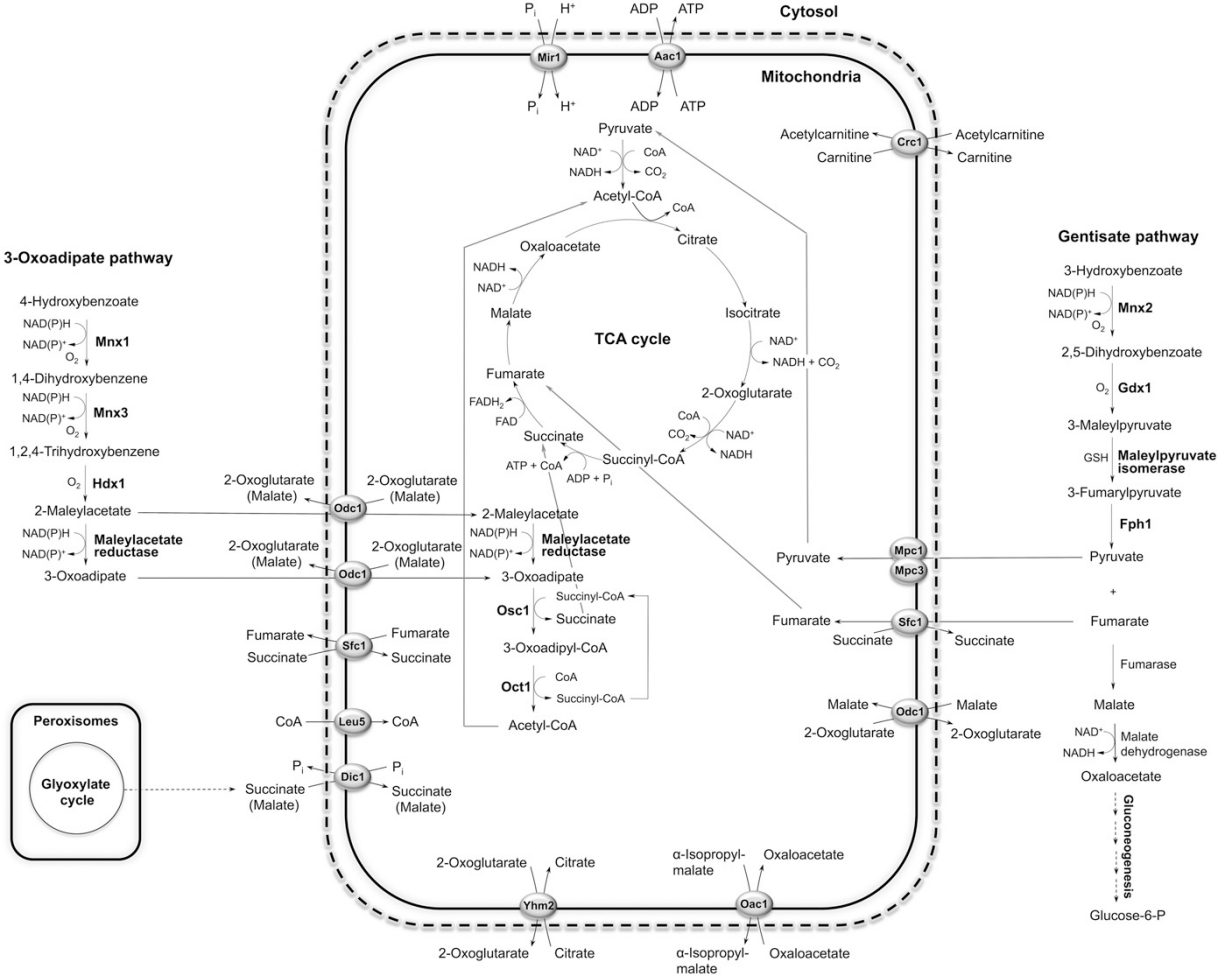
Proposed integration of the 3-OAP and GP with mitochondrial metabolism through MC proteins. The enzymes of the 3-OAP and GP are highlighted with bold letters. Enzyme abbreviations: Mnx1, 4-hydroxybenzoate 1-hydroxylase; Mnx3, hydroquinone hydroxylase; Hdx1, hydroxyquinol 1,2-dioxygenase; Osc1, 3-oxoadipate CoA-transferase; Oct1, 3-oxoadipyl-CoA thiolase; Mnx2, 3-hydroxybenzoate 6-hydroxylase; Gdx1, gentisate 1,2-dioxygenase; Fph1, fumarylpyruvate hydrolase. Carrier acronyms: Odc1, oxodicarboxylate carrier; Sfc1 succinate/fumarate carrier; Leu5, CoA carrier; Dic1, dicarboxylate carrier; Yhm2, oxoglutarate/citrate carrier; Oac1, oxaloacetate/isopropylmalate carrier; Mpc1 and 3, subunits of pyruvate carrier; Crc1, carnitine carrier; Aac1, ADP/ATP carrier; Mir1, phosphate carrier; GSH, glutathione.

The proposed integration of the 3-OAP and GP with mitochondrial metabolism via biochemical reactions, and transport of metabolites across the inner mitochondrial membrane, is shown in Figure 6. The first three reactions of the 3-OAP, fulfilled by Mnx1p, Mnx3p, and Hdx1p, proceed in the cytoplasm (Holesova *et al.* 2011; Figure 2). The fourth reaction is catalyzed by maleylacetate reductase, but the protein and corresponding gene have not been identified so far. The last two reactions, performed by Osc1p and Oct1p, are localized inside mitochondria (Figure 2), which implies that either 2-maleylacetate, or 3-oxoadipate, is transported into the organelle. Both intermediates are six-carbon oxodicarboxylates, and could be transported via oxodicarboxylate carrier. In *S. cerevisiae*, Odc1p and Odc2p have been identified as high affinity transporters of C_5_–C_7_ (oxo)dicarboxylates (Palmieri *et al.* 2001). By inspecting the genome of *C. parapsilosis*, we found Odc1p as the only isoform of oxodicarboxylate carrier. The expression of *ODC1* gene is not repressed by glucose, similar to *Sc**ODC2*. Thus, 3-oxoadipate (or 2-maleylacetate) is likely imported into mitochondria in exchange for 2-oxoglutarate (or malate). Similar antiport of 2-oxoadipate *vs.* 2-oxoglutarate (or malate) has been proposed as relevant transport mode in *S. cerevisiae* (Palmieri *et al.* 2006).

Another important role of *Odc1p* is probably 2-oxoglutarate/malate antiport as part of the malate/aspartate shuttle, and to provide 2-oxoglutarate for cytosolic conversion into glutamate and for nitrogen assimilation as described for *S. cerevisiae* (Palmieri *et al.* 2006). *Sc*Odc1p and *Sc*Odc2p are necessary for growth on oleic acid (Tibbetts *et al.* 2002), and *Sc*Ymc1p and *Sc*Ymc2p were identified as suppressors of the oleic acid growth defect of the *S. cerevisiae* double mutant Δ*odc1*Δ*odc2*. *Sc*Ymc1p and *Sc*Ymc2p are also important for glutamate biosynthesis, which is explained by possible overlapping transport activity with *Sc*Odc1p and *Sc*Odc2p (Trotter *et al.* 2005). In *C. parapsilosis*, we detected a minor increase of *YMC1* and *YMC2* expression by 3OH, while only the *YMC1* gene was induced by 4OH (Figure 3 and Table S6). We hypothesize that Ymc1p and Ymc2p can at least partially substitute the functions in metabolism that we assigned to Odc1p, namely 2-oxoglutarate/malate antiport. Ymc1p could be involved also in the import of 3-oxoadipate during catabolism of 4OH.

Enzymes Mnx2p and Gdx1p, performing the first two reactions of the GP, colocalize with mitochondria in cells assimilating 3OH or gentisate, but not when the cells are grown in glucose medium (Holesova *et al.* 2011). Moreover, they apparently do not contain a mitochondrial import presequence. Therefore, it is unlikely that these proteins reside in mitochondria. Rather, their mitochondrial association could result, for example, from interaction with an unidentified protein present in outer mitochondrial membrane when yeast cells metabolize hydroxybenzoates. Subcellular localization of maleylpyruvate isomerase, the third enzyme of the GP, is unknown, as the corresponding gene remains elusive. However, fumarylpyruvate hydrolase, the last enzyme of the GP, is localized in cytosol (Figure 2). This enzyme catalyzes the hydrolytic cleavage of fumarylpyruvate to fumarate and pyruvate, which can be transported into mitochondria via MC proteins. By analogy to *S. cerevisiae* (Bricker *et al.* 2012; Herzig *et al.* 2012), pyruvate is likely imported into mitochondria of *C. parapsilosis* by a pyruvate carrier consisting of a Mpc1/Mpc3 heterodimer, and our results show that both *MPC1* and *MPC3* genes in *C. parapsilosis* are induced in cells grown in S3OH medium.

*SFC1* is the most induced MC gene, in both S3OH and S4OH media (Figure 3 and Table S6). *Sc*Sfc1p is the only succinate/fumarate carrier characterized by transport assays of purified protein in liposomes. It transports succinate and fumarate with the highest affinities, and, to a lesser extent, also oxoglutarate, oxaloacetate, malate, *cis*-aconitate, and isocitrate (Palmieri *et al.* 1997). Hence, the Sfc1 carrier can import fumarate into mitochondria via antiport with succinate due to the abundance of fumarate produced by Fph1p in the cytosol. Another option is import of fumarate via fumarate/malate antiport described in *S. cerevisiae* mitochondria (Pallotta *et al.* 1999). A fraction of fumarate can be metabolized directly in the cytoplasm to yield malate, which is transported into mitochondria via Odc1p, or is further converted to oxaloacetate, which enters gluconeogenesis. High level of Sfc1p expression during metabolism of 4OH is likely connected to succinate/fumarate antiport, when intramitochondrial fumarate from the TCA cycle is exported to the cytoplasm to participate in gluconeogenesis, and succinate originated in glyoxylate cycle is imported into mitochondria, as postulated for *Sc*Sfc1p (Palmieri *et al.* 2006).

*DIC1* is expressed in media containing hydroxybenzoates, but to a lower level than *SFC1* gene. Dic1p probably does not transport molecules generated in the 3-OAP and GP, as it does not exchange fumarate, and its main function is to import malate, succinate, and malonate into mitochondria in exchange for phosphate (Palmieri *et al.* 1996). We suppose that Dic1p supplies the TCA cycle with cytoplasmic dicarboxylates, *e.g.*, succinate or malate produced in the glyoxylate cycle, like in *S. cerevisiae* (Palmieri *et al.* 1999c).

*YHM2* is induced ∼3-fold in both S3OH and S4OH media, indicating its importance for cell metabolism. In *S. cerevisiae*, Yhm2p associates with mitochondrial nucleoids *in vivo* and supports growth on nonfermentable carbon sources (Cho *et al.* 1998). *Sc*Yhm2p exports citrate from mitochondria by 2-oxoglutarate/citrate antiport. Citrate is converted through isocitrate to 2-oxoglutarate by NADP^+^ dependent isocitrate dehydrogenase, increasing thus NADPH reducing power in the cytosol required for biosynthetic and antioxidant reactions (Castegna *et al.* 2010). As both the 3-OAP and GP consume NAD(P)H in the cytosol (Figure 6), activity of Yhm2p could contribute to replenishment of this cofactor.

Oac1p is not induced significantly by hydroxyaromatic substrates, but a relatively high level of expression has been determined in cells grown in both S3OH and S4OH (Figure 3). Oac1p is probably not involved in transport of final products of the GP and 3-OAP, as it transports pyruvate, fumarate, and succinate very poorly. It rather supplies the TCA cycle with intermediates, as import of oxaloacetate into mitochondria was proposed as the physiologically relevant role of Oac1p (Palmieri *et al.* 1999b). Oxaloacetate can also be converted to pyruvate, which is a substrate for the first step in leucine and valine biosynthesis (Boer *et al.* 2005). Oac1p-mediated export of mitochondrial α-isopropylmalate, probably by antiport with oxaloacetate, is important for leucine biosynthesis in the cytosol (Marobbio *et al.* 2008).

Crc1p is involved in carnitine-dependent transport of acetyl-CoA from peroxisomes to mitochondria during fatty acid β-oxidation, when acetylcarnitine is exchanged for carnitine (Palmieri *et al.* 1999a; van Roermund *et al.* 1999). This function of Crc1p is anticipated also for intracellular acetyl unit transport in the yeast *C. albicans* (Strijbis and Distel 2010). *CRC1* encoding a carnitine carrier has the lowest level of expression in media with hydroxybenzoates from all MCs tested by RT-qPCR (Figure 3). The level of *CRC1* expression is higher in cells grown in S3OH medium than in S4OH medium. The lower demand for Crc1p-mediated import of acetyl-CoA into mitochondria during utilization of 4OH can be rationalized by generation of acetyl-CoA from 3-oxoadipyl-CoA in the reactions of the 3-OAP (Figure 6). *AAC1* and *MIR1* are two genes with the highest expression levels among genes for MCs when cells are grown in S3OH or S4OH media (Table S3). The significance of both carriers for cell metabolism results from their essential role for intramitochondrial synthesis of ATP.

*Leu5p* is required for accumulation of CoA in the mitochondrial matrix, thus contributing to performance of intramitochondrial reactions dependent on CoA (Prohl *et al.* 2001). CoA imported into mitochondria is utilized in the TCA cycle and heme biosynthesis. *LEU5* is induced in media with hydroxybenzoates exhibiting higher induction in S4OH than in S3OH (Figure 3, Table S3, and Table S6) suggesting higher demand for CoA inside mitochondria of cells grown in media with 4OH, which can be explained by an additional intramitochondrial reaction consuming CoA, such as the last step of the 3-OAP (Figure 6).

### The 3-OAP and GP have different evolutionary trajectories

**Figure 7:**
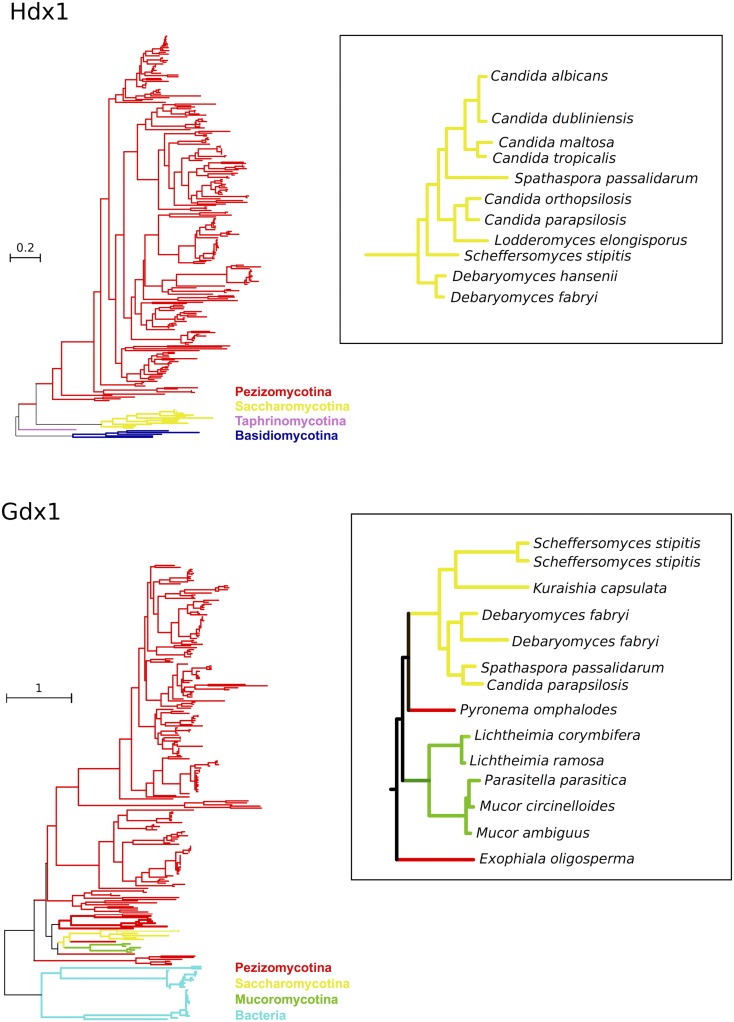
Molecular phylogenies of proteins encoded by *HDX1* and *GDX1* genes. The sequences of key enzymes of the 3-OAP and GP, *i.e.*, Hdx1 (upper panel) and Gdx1 (lower panel), respectively, were used for constructing the phylogenies. The whole tree structure is shown schematically, with lineages colored according to their taxonomic classification: Pezizomycotina in red, Basidiomycotina in blue, Mucoromycotina in green, Saccharomycotina in yellow, Taphrinomycotina in pink, and Bacteria in cyan. Saccharomycotina-containing subtrees are shown in more detail in the corresponding insets, to show the name of the species.

**Figure 8:**
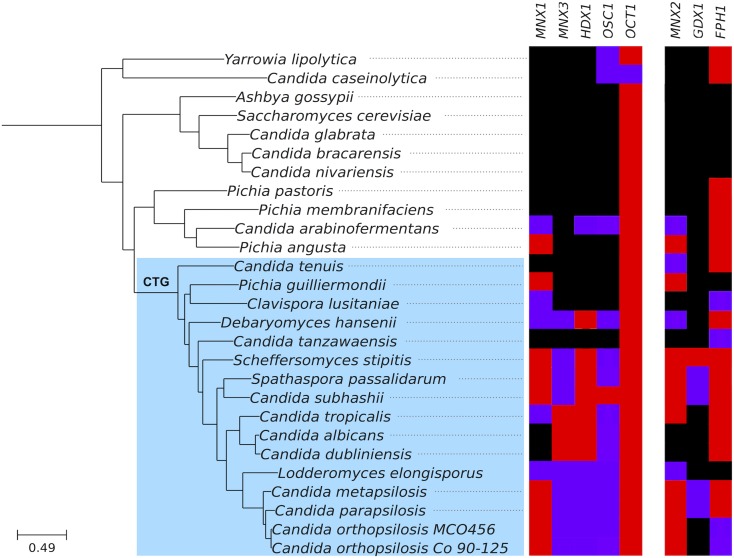
Phylogenetic profile showing the presence and absence of genes of the 3-OAP (left panel) and GP (right panel) across several sequenced Saccharomycotina species. The species phylogeny has been adapted from Gerecova *et al.* (2015). Presence and absence profiles are based on results from a Blast search (*E*-value < 10^−25^), which are indicated with the following colors: black (no homolog), violet (exactly one homolog), and red (two or more homologs).

To gain insight into the evolution and genetic distribution of 3-OAP and GP, we used a phylogenomic approach consisting of searching for homologs in a set of fully sequenced genomes, inspecting available phylogenies in PhylomeDB (Huerta-Cepas *et al.* 2014), and reconstructing evolutionary trajectories for each of the genes in the pathway (see *Materials and Methods*). Genes in both pathways presented a very sparse distribution across sequenced Saccharomycotina, but were widespread among Pezizomycotina species (Figure 7). More specifically, the 3-OAP seems to be complete, or almost complete, in the following CTG clade species beyond *C. parapsilosis*: *C. albicans*, *C. dublinensis*, *C. maltosa*, *C. orthopsilosis*, *C. tropicalis*, *D. hansenii*, *D. fabryi*, *Lodderomyces elongisporus*, *S. stipitis*, and *Spathaspora passalidarum*. The presence of the GP seems to be more restricted among Saccharomycotina, being complete only in *C. parapsilosis*, *D. fabryi*, *S. stipitis*, *S. passalidarum*, and, surprisingly, the non-CTG clade species *Kuraishia capsulata*. Homologs of some genes of the two pathways (mostly *FPH1*, *OCT1*, and *OSC1*) were present in isolation in some other species, but the absence of the other counterparts suggests they may perform alternative functions (Figure 8).

Inspection of the phylogenies of 3-OAP genes showed that, with few exceptions, they conformed to the known species tree of the Saccharomycotina, suggesting vertical descent within this clade. Phylogenies showed Saccharomycotina sequences branching next to large clades formed by Pezizomycotina sequences (the sister group of Saccharomycotina). This pattern is compatible either with ancient transfer from a Pezizomycotina ancestor to an ancient lineage in the CTG clade, or by simple vertical descent from their common ancestor and subsequent losses in non-CTG lineages. Such patterns of differential gene loss are not rare in Saccharomycotina (Morel *et al.* 2015). In addition, we observed long branches separating the Saccharomycotina sequences from their Pezizomycotina counterparts. Considering all this, and the abovementioned presence of orthologous of some of these genes in a few early branching Saccharomycotina such as *OSC1* in *Yarrowia lipolytica* and *Geotrichum candidum*, we favor the vertical inheritance hypothesis.

The phylogenies of the genes from the GP, however, revealed patterns more difficult to reconcile with scenarios involving vertical descent only. The three genes *MNX2*, *GDX1*, and *FPH1* show striking phylogenetic affiliations with genes from the distantly related Mucorales species belonging to zygomycete fungi. Although these phylogenetic relationships are not highly supported, they come up consistently with different phylogenetic approaches. In addition, inspection of the gene localization of these genes in the Mucorales genome showed they are in the vicinity of each other, suggesting they also form a cluster in Mucorales. Thus a plausible scenario involves transfers between an ancestor of currently sequenced Mucorales and an ancestor of the Saccharomycotina carrying this cluster. Considering that the Mucorales and the CTG clade species are sister groups to Pezizomycotina species, a transfer from Ascomycetes to Mucorales seems more plausible than the scenario involving alternative directionality.

Finally, in an attempt to identify additional players in the 3-OAP and GP pathways, we searched for genes in *C. parapsilosis* showing similar phylogenetic distributions (see *Materials and Methods*). We provide lists of the top 372 genes showing a higher degree of coevolution with the key members of each pathway (*i.e.*, *HDX1* and *GDX1*), and whose expression is induced upon exposure to the main substrate catalyzed by each pathway (Table S9). This approach revealed that a candidate coding for maleylacetate reductase functioning in the 3-OAP is CPAR2_406430, which has a relatively similar phylogenetic profile to genes in this pathway (Hamming distance of 15 to the Hdx1 profile), and is located in the vicinity of the 3-OAP gene cluster, upstream of the *HDX1* gene (Gerecova *et al.* 2015). CPAR2_406430 encodes a putative flavin reductase-like protein containing an FMN-binding domain. Importantly, it is upregulated (102-fold) in S4OH medium (Table S5). An obvious candidate for functioning in the GP is CPAR2_704360 (Hamming distance of 14 to the Gdx1 profile), which encodes a putative protein with carbon-sulfur lyase activity, and a domain of glutathione-dependent formaldehyde-activating enzyme. This gene is adjacent to the GP gene cluster, downstream of *FPH1* (Holesova *et al.* 2011), and is highly induced (1402-fold) in S3OH medium (Table S5). We speculate that it may represent the missing GP gene coding for maleylpyruvate isomerase, which isomerizes 3-maleylpyruvate to 3-fumarylpyruvate in a glutathione-dependent reaction. An additional candidate is CPAR2_704370 (Hamming distance of 20 to the Gdx1 profile), which is induced about 10-fold upon exposure to S3OH (Table S5). This gene is also located close to the GP gene cluster, and it encodes a putative transcription factor. Importantly, a homolog of this gene in Mucorales is also placed in the vicinity of the GP genes. Moreover, our preliminary data indicate that a *C. parapsilosis* mutant lacking this gene does not grow on S3OH medium (A. Cillingová, R. Tóth, A. Gácser, and J. Nosek, unpublished results), strongly supporting its role as a transcriptional regulator of the GP. However, future research is needed to confirm the role of CPAR2_704370, CPAR2_704360, and CPAR2_406430 in the metabolism of hydroxybenzoates.

### Conclusions

In this study, we investigated the connection of two metabolic pathways involved in the catabolism of hydroxybenzoates to mitochondria in *C. parapsilosis*. We determined that genes encoding enzymes of the 3-OAP and GP are highly induced by the corresponding hydroxybenzoate substrate, and several enzymes of these pathways associate with mitochondria. Our data show that both pathways are linked to mitochondrial metabolism through the produced metabolites. This functional connection is mediated via MC proteins such as succinate/fumarate carrier, which are highly expressed, and/or selectively induced, in cells assimilating hydroxybenzoates. Our results imply that mitochondrial carriers are transporting distinct intermediates, final products of the 3-OAP and GP, or other metabolites to supply mitochondria mainly with substrates for the TCA cycle and oxidative phosphorylation, and the cytosol with products of intramitochondrial reactions. In addition, we have found that these pathways are sparsely distributed among Saccharomycotina, having been lost in numerous lineages of this clade. This patchy distribution allows us to prioritize a set of candidate genes whose function may be related to each of these metabolic pathways. Interestingly, we identified possible ancient events of horizontal gene transfer of the GP genes to the distant Mucorales. The 3-OAP and GP thus represent interesting examples of two related biochemical pathways with distinct evolutionary histories.

## Supplementary Material

Supplemental Material
