# Supplementary material for: Mitochondrial Carriers Link the Catabolism of Hydroxyaromatic Compounds to the Central Metabolism in Candida parapsilosis
Source: G3 (Bethesda). 2016 Oct 3;6(12):4047–58. doi: 10.1534/g3.116.034389 (PMC5144973; doi:10.1534/g3.116.034389)
Supplement: Supplemental Material [file supp_6_12_4047__index.html]

Mitochondrial Carriers Link the Catabolism of Hydroxyaromatic Compounds to the Central Metabolism in Candida parapsilosis — Supplemental Material 

# Mitochondrial Carriers Link the Catabolism of Hydroxyaromatic Compounds to the Central Metabolism in *Candida parapsilosis*

## Supplemental Material for Zeman, *et al*, 2016

**Files in this Data Supplement:**

- Figure S1 - The unrooted phylogenetic tree of the MCF proteins in *C. parapsilosis*. (.pdf, 216 KB)
- Figure S2 - Amino acid sequence alignment of the Sfc1 orthologs. (.pdf, 3,917 KB)
- Table S1 - Oligonucleotides used in this study. (.xlsx, 32 KB)
- Table S2 - Plasmid constructs used in this study. (.xlsx, 42 KB)
- Table S3 - Expression of the genes investigated in this study by RNA-seq analysis. (.xlsx, 57 KB)
- Table S4 - Sequencing and alignment statistics. (.xlsx, 49 KB)
- Table S5 - Expression data obtained by RNA-seq analysis. (.xlsx, 622 KB)
- Table S6 - Expression data obtained by RT-qPCR quantification of the *C. parapsilosis* genes involved in the 3-OAP (*MNX1, MNX3, HDX1, OSC1, OCT1*), the GP (*MNX2, GDX1, FPH1*) and the genes encoding MCs (*CRC1, DIC1, LEU5, MPC1, MPC3, OAC1, ODC1, SFC1, YHM2, YMC1, YMC2*). (.xlsx, 64 KB)
- Table S7 - Intracellular localization of the 3-oxoadipate and gentisate pathway enzymes. (.xlsx, 38 KB)
- Table S8 - Mitochondrial carriers in *C. parapsilosis*. (.xlsx, 44 KB)
- Table S9 - Phylogenetic profiles of *C. parapsilosis* proteins - (A) Hdx1. (.xlsx, 75 KB)
