# Supplementary material for: Mitochondrial Carriers Link the Catabolism of Hydroxyaromatic Compounds to the Central Metabolism in Candida parapsilosis
Source: G3 (Bethesda). 2016 Oct 3;6(12):4047–58. doi: 10.1534/g3.116.034389 (PMC5144973; doi:10.1534/g3.116.034389)
Supplement: Supplemental Material [file supp_g3.116.034389_FigureS1.pdf]

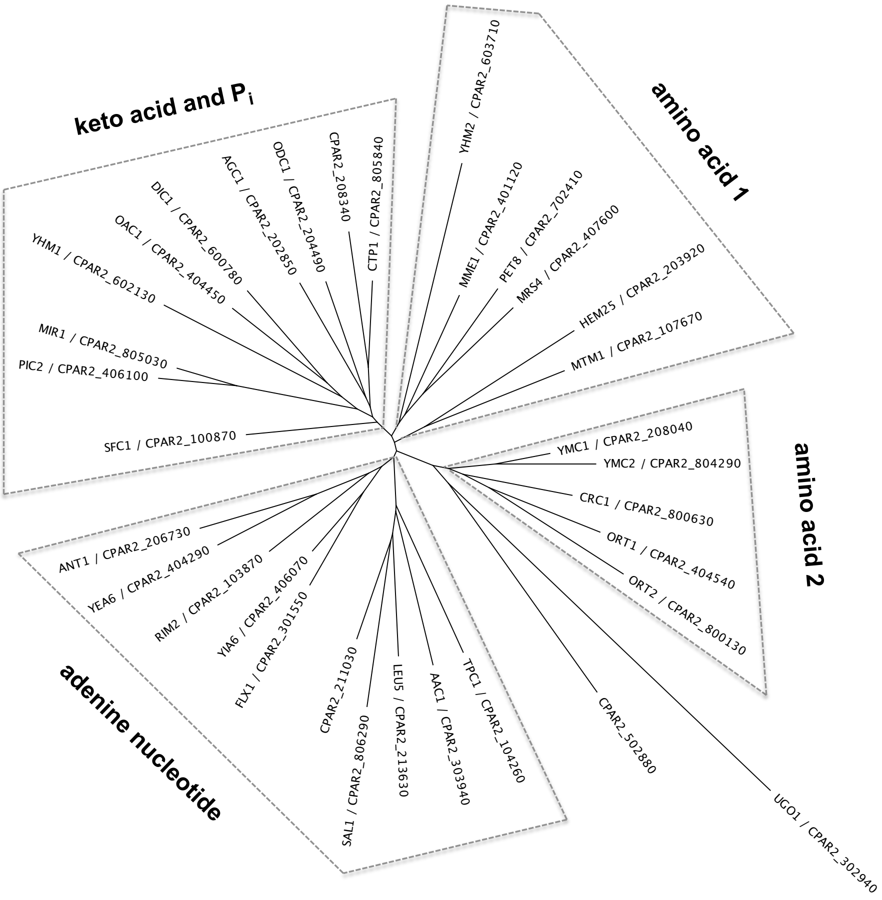

Figure S1 The unrooted phylogenetic tree of the MCF proteins in *C. parapsilosis*. The tree was calculated by maximum likehood method (Guindon and Gascuel 2003) with Geneious plugin PhyML v. 2.1.0. MCs are distributed into four groups based on substrate structure. Proteins form group of adenine nucleotide carriers, group of keto acid and phosphate carriers and two groups of amino acid carriers. Distribution of proteins into groups is consistent with the categorization based on the transported substrates with few exceptions. CPAR2_202850, the homolog of aspartate/glutamate carrier *Sc*Agc1p (Cavero *et al*. 2003) clusters with keto acid and phosphate subfamily and CPAR2_603710, the homolog of oxoglutarate/citrate carrier *Sc*Yhm2p (Castegna *et al*. 2010) clusters with amino acid group 1. *Candida* specific protein CPAR2_502880 clusters with homolog of *Sc*Ugo1p, which is localized in the outer mitochondrial membrane and is engaged in mitochondrial fusion (Sesaki and Jensen 2001; Hoppins *et al*. 2009).

Supplemental References:

1. Castegna, A., P. Scarcia, G. Agrimi, L. Palmieri, H. Rottensteiner *et al.*, 2010 Identification and functional characterization of a novel mitochondrial carrier for citrate and oxoglutarate in *Saccharomyces cerevisiae*. J. Biol. Chem. 285: 17359–17370.
2. Cavero, S., A. Vozza, A. del Arco, L. Palmieri, A. Villa *et al.*, 2003 Identification and metabolic role of the mitochondrial aspartate-glutamate transporter in *Saccharomyces cerevisiae*. Mol. Microbiol. 50: 1257–1269.
3. Guindon, S., and O. Gascuel, 2003 A simple, fast, and accurate algorithm to estimate large phylogenies by maximum likelihood. Syst. Biol. 52: 696–704.
4. Hoppins, S., J. Horner, C. Song, J. M. McCaffery, and J. Nunnari, 2009 Mitochondrial outer and inner membrane fusion requires a modified carrier protein. J. Cell Biol. 184: 569–581.
5. Sesaki, H., and R. E. Jensen, 2001 *UGO1* encodes an outer membrane protein required for mitochondrial fusion. J. Cell Biol. 152: 1123–1134.
