# Supplementary material for: Mitochondrial Carriers Link the Catabolism of Hydroxyaromatic Compounds to the Central Metabolism in Candida parapsilosis
Source: G3 (Bethesda). 2016 Oct 3;6(12):4047–58. doi: 10.1534/g3.116.034389 (PMC5144973; doi:10.1534/g3.116.034389)
Supplement: Supplemental Material [file supp_g3.116.034389_FigureS2.pdf]

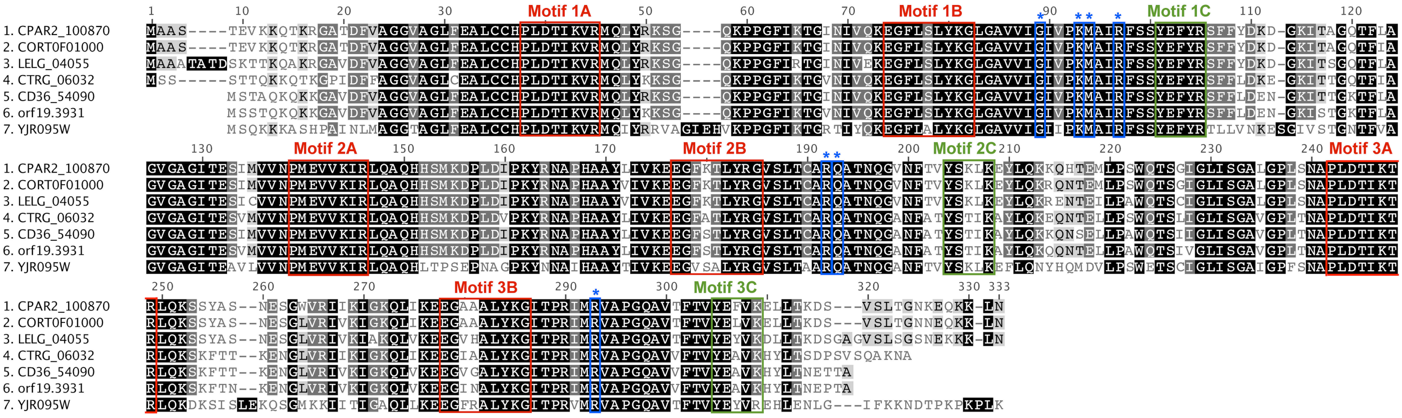


**Figure S2** Amino acid sequence alignment of the Sfc1 orthologs. The sequences are from the following yeast species: *C. parapsilosis* (CPAR2_100870), *C. orthopsilosis* (CORT0F01000), *L. elongisporus* (LELG_04055), *C. tropicalis* (CTRG_06032), *C. dubliniensis* (CD36_54090), *C. albicans* (orf19.3931) and *S. cerevisiae* (YJR095W). All motifs are marked with rectangles. Residues involved in substrate binding present in contact points I (G, KM, R), II (RQ) and III (R) (Kunji and Robinson 2006) are marked with rectangles and asterisks. MCs contain six transmembrane helices connected by three matrix and two cytosolic loops. The proteins have three similar domains, each with size approximately 100 amino acids. Sfc1p contains three such domains (PFAM00153), at amino acid positions 16-103, 107-205 and 213-304. Each domain bears the 'mitochondrial energy transfer signature' motif. First part of the motif P-h-[D/E]-X-h-[K/R]-X-[R/K], where h represents a hydrophobic residue, is in the C-terminal part of odd helices 1, 3 and 5 (motifs 1A, 2A and 3A). After spacer of 20 - 30 amino acids there is the second part [D/E]-G-$X_4$-[W/Y/F]-[K/R]-G (motifs 1B, 2B and 3B), which is present at the end of matrix loops 1, 2 and 3 and continues in the N-terminal part of even helices 2, 4 and 6 (Palmieri 2004; Palmieri and Pierri 2010). The sequences of the *C. parapsilosis* Sfc1p (CPAR2_100870) signature motifs match perfectly with the consensus sequence, and they are identical with *Sc*Sfc1p signature sequences at the level of individual residues. Few differences are present only within degenerated sequences $X_4$. Conserved residues present in three P-h-[D/E]-X-h-[K/R] sequences, which are the subset of the signature motifs, are required for transport. Charged residues form a salt bridge network on the matrix side of the protein cavity when the substrate-binding site is open to the mitochondrial intermembrane space. Functionally similar motifs [F/Y]-[D/E]-X-X-[R/K] which are parts of the even-numbered helices 2, 4 and 6 are present on the cytoplasmic side of the cavity and could form a salt bridge network when the substrate-binding site is accessible from the mitochondrial matrix (Robinson *et al*. 2008). This motif (motifs 1C, 2C and 3C) is preserved in both Sfc1 homologs in helices 2, 4 and 6 with one change in sequence YSKLK in helix 4 where polar residue serine is present instead of negatively charged residue. Substrate binding site present in the central part of *Sc*Sfc1p protein cavity consists of residues constituting three contact points I, II and III in transmembrane helices 2, 4 and 6, respectively (Kunji and Robinson 2006). Residues in contact points I (G, KM, R), II (RQ) and III (R) are identical in the Sfc1 proteins from *S. cerevisiae* and *C. parapsilosis*.

**Supplemental References:**


1. Kunji, E. R., and A. J. Robinson, 2006 The conserved substrate binding site of mitochondrial carriers. Biochim. Biophys. Acta 1757: 1237–1248.
2. Palmieri, F., 2004 The mitochondrial transporter family (SLC25): physiological and pathological implications. Pflugers Arch. 447: 689–709.
3. Palmieri, F., and C. L. Pierri, 2010 Structure and function of mitochondrial carriers - role of the transmembrane helix P and G residues in the gating and transport mechanism. FEBS Lett. 584: 1931–1939.
4. Robinson, A. J., C. Overy, and E. R. Kunji, 2008 The mechanism of transport by mitochondrial carriers based on analysis of symmetry. Proc. Natl. Acad. Sci. U.S.A. 105: 17766–17771.
